# Transcriptome analysis of hormone-induced gene expression in *Brachypodium distachyon*

**DOI:** 10.1038/srep14476

**Published:** 2015-09-30

**Authors:** Yusuke Kakei, Keiichi Mochida, Tetsuya Sakurai, Takuhiro Yoshida, Kazuo Shinozaki, Yukihisa Shimada

**Affiliations:** 1 Kihara Institute for Biological Research, Yokohama City University, Kanagawa, JAPAN; 2 Cellulose Production Research Team, Biomass Engineering Program Cooperation Division, RIKEN Center for Sustainable Resource Science, Kanagawa, JAPAN; 3 Integrated Genome Informatics Research Unit, RIKEN Center for Sustainable Resource Science, Kanagawa, JAPAN; 4 Gene Discovery Research Group, RIKEN Center for Sustainable Resource Science, Kanagawa, JAPAN; 5 Biomass Research Platform Team, Biomass Engineering Research Division, RIKEN Center for Sustainable Resource Science, Kanagawa, JAPAN

## Abstract

*Brachypodium distachyon* is a new model plant closely related to wheat and other cereals. In this study, we performed a comprehensive analysis of hormone-regulated genes in *Brachypodium distachyon* using RNA sequencing technology. *Brachypodium distachyon* seedlings were treated with eight phytohormones (auxin, cytokinine, brassinosteroid, gibberelline, abscisic acid, ethylene, jasmonate and salicylic acid) and two inhibitors, Brz220 (brassinosteroid biosynthesis inhibitor) and prohexadione (gibberelline biosynthesis inhibitor). The expressions of 1807 genes were regulated in a phytohormone-dependent manner. We compared the data with the phytohormone responses that have reported in rice. Transcriptional responses to hormones are conserved between *Bracypodium* and rice. Transcriptional regulation by brassinosteroid, gibberellin and ethylene was relatively weaker than those by other hormones. This is consistent with the data obtained from comprehensive analysis of hormone responses reported in *Arabidopsis*. *Brachypodium* and *Arabidopsis* also shared some common transcriptional responses to phytohormones. Alternatively, unique transcriptional responses to phytohormones were observed in *Brachypodium*. For example, the expressions of ACC synthase genes were up-regulated by auxin treatment in rice and *Arabidopsis*, but no orthologous ACC synthase gene was up-regulated in *Brachypodium*. Our results provide information useful to understand the diversity and similarity of hormone-regulated transcriptional responses between eudicots and monocots.

Human diets greatly depend on grasses, which have applications as energy crops and sustainable energy sources. Rice (*Oryza sativa*) and maize (*Zea mays*) are commonly used as model plants for monocots, as their genome sequence and extensive genetic resources are available^1^. Draper *et al.* proposed *Brachypodium* (*Brachypodium distachyon*) as a novel model plant in 2001 because it has advantages such as a small genome size, short life cycle, small plant size, and the ability to self-pollinate^2^. Furthermore, *Brachypodium* is more closely related to wheat [*Triticum aestivum*] and rye [*Secale cereale*] than rice and maize are, which makes it convenient for analysis of different grass groups using model systems^1^. The whole genome sequence of *Brachypodium* inbred line Bd21 was released in 2010 as the first genome sequence available for the Pooideae subfamily^3^. Recently, a comprehensive collection of full-length cDNAs was reported by Mochida *et al.*^4^. Therefore, fundamental resources for *Brachypodium* molecular biology are being rapidly developed. Recently, Priest *et al.* reported an analysis of global gene expression in *Brachypodium* in response to abiotic stress^5^. Large-scale gene expression data are essential resources for model systems. For *Arabidopsis*, The Arabidopsis Information Resource (TAIR: http://www.arabidopsis.org) and Arabidopsis e-FP browser^6^ integrate the public transcriptome data with other information to provide information useful for analysis of gene functions. Many tools for biological data mining, such as co-expression analysis (e.g., ATTED-II^7^), transcriptome network analysis (AtCAST^8^,^9^), and prediction of transcriptome dynamics in natural conditions^10^, depend on the availability of large-scale transcriptome data.

Plant growth and development depend on phytohormone-mediated regulation of gene expression. Auxins, cytokinins (CKs), gibberellins (GAs) and brassinosteroids (BRs) generally promote plant growth and greatly influence plant stature and organ size. In contrast, ethylene, abscisic acid (ABA), salicylic acid (SA) and jasmonate (JA) regulate stress-related responses and/or growth retardation. The comprehensive response to a hormone was first reported in the eudicot model plant *Arabidopsis thaliana* by the AtGenExpress project (http://atpbsmd.yokohama-cu.ac.jp)^11^. Hundreds of genes have been identified as being regulated by phytohormones. These comprehensive transcriptome data have been utilized in hundreds of studies of large-scale or gene-specific regulation of transcripts. Therefore, analyses of phytohormone-regulated transcriptomes in *Brachypodium* are essential for transcriptomic and genetic studies of this plant. *Brachypodium* shares about 80% of its genes with rice as homologs^12^, and is more closely related to rice than to *Arabidopsis* and other model plants. Analyses of phytohormone-regulated transcriptomes were performed in rice for auxin, CK, SA, ABA, JA, and ethylene^13^ and for auxin, CK, GA, BR, ABA, and JA^14^. Comparisons of the responses in *Brachypodium* and these other model plants will reveal both responses common to all plants and those specific to Pooideae.

GA and BR treatment generates almost no change in gene expression in wild-type *Arabidopsis* seedlings^11^; indeed, the endogenous GA and BR levels are too high (i.e., saturated) for exogenous hormones to induce gene expression. Therefore, BR- and GA-regulated genes in *Arabidopsis* have been identified using the BR- and GA-deficient mutants *det2* and *ga-1*, respectively, and treatment with brassinolide (BL; BR), GA_3_, or GA_4_^11^. In this study, we found that the transcriptional regulation of BR-regulated genes in BR-treated *det2* plants was negatively correlated with BR-biosynthesis inhibitor treatment using the AtCAST database (http://atpbsmd.yokohama-cu.ac.jp/atcast/html/genelist/A0036_A0081.html)^9^. These findings suggest that the application of hormone biosynthesis inhibitors (Brz220 for BR and Phx for GA) to generate hormone-deficient conditions is a practical means of identifying BR- or GA-regulated genes in *Brachypodium*.

In this study, we performed a comprehensive RNA-seq analysis of the transcriptional responses in *Brachypodium* to eight phytohormones; auxin, BR, CK, GA, SA, ABA, JA, and ethylene. The compounds assayed included indole-3-acetic acid (IAA; auxin), trans-zeatin (tZ; CK), BL, Brz220 (Brz; inhibitor of BR synthesis), GA_4_, prohexadione-calcium (Phx: inhibitor of GA synthesis), SA, ABA, methyl jasmonate (MJ; JA) and 1-amino-cyclopropane-1-carboxylic acid (ACC; ethylene precursor). In addition, we applied combination treatments of BL and Brz220 (a BR-biosynthesis inhibitor) and GA4 and Phx (GA-biosynthesis inhibitor) to *Brachypodium*. Transcriptional regulation of genes in *Brachypodium* was compared to that in Rice and *Arabidopsis thaliana*.

## Results

### Determination of hormone treatment conditions and identification of hormone-regulated genes

We selected phytohormone-responsive marker genes in *Brachypodium* using the following procedure. Twenty-eight phytohormone-responsive genes from rice^13^ and *Arabidopsis thaliana*^11^,^15^ were selected and their sequences were subjected to a BLAST search for the most similar sequences in *Brachypodium*. Top-hit *Brachypodium* genes were used to examine transcriptional responses in *Brachypodium* (Table 1). We checked the transcriptional responses with RT-PCR. In total, 19 of 28 genes showed reproducible gene expression responses to one of the phytohormones or the inhibitors in more than two independent experiments. These 19 genes were selected as phytohormone-responsive marker genes in *Brachypodium* (Table 1). Next, hormone treatment conditions in *Brachypodium* were determined using these marker genes. We used 1, 3, and 10 μM for IAA, tZ and GA; 10, 30, and 100 μM for Phx, ABA, MJ, ACC, Brz220 and SA; and 1, 10, 100, and 1000 μM for BL. The concentrations of phytohormones and inhibitors used in the comprehensive transcriptome analysis were determined accordingly. The optimum concentration of each phytohormone (Table 2) was determined as that at which the marker gene showed the greatest change in response to treatment compared to mock treatment. After the determination of treatment conditions, the hormone treatment and sampling were repeated twice independently and the hormonal responses were confirmed against the transcriptional responses of the marker genes. Total RNAs extracted in two independent experiments were mixed and used in RNA-seq analysis.

About 90% of obtained reads from the RNA-seq analysis were successfully mapped to the *Brachypodium* genome (Table 3). Differentially expressed genes (DEGs) in response to each treatment (FDR < 0.05) were analyzed using edgeR^16^ in a comparison with all other treatments (Table 4). Hundreds of genes were differentially expressed in response to ABA and MJ. Dozens of DEGs were detected for the IAA, tZ and SA treatments. We designated these analyses as being high stringency; the hormone-responsive genes are listed in Data S1–S8. In contrast, only four genes were differentially expressed in the Brz220 treatment. No DEG was detected in the mock 1-h, mock 3-h, Phx, Phx+GA, Brz220+BL and ethylene treatments, indicating that the gene expression responses to GA, BR and ethylene were weaker than those to the other hormones. Therefore, we conducted additional statistical analyses to identify the genes responsive to these hormones. For these analyses, DEGs were compared between the hormone treatment vs. two control treatments (Table 5). Two hundred and twenty-four DEGs were detected in response to GA, 474 DEGs in response to BR, and 52 DEGs in response to ethylene. We designated these analyses as low stringency; the DEGs are listed in Table 4, Data S9–S16.

### Comparison with rice and Arabidopsis

First, we compared the phytohormone-regulated genes in *Brachypodium* with those in rice. A comparison of our experimental conditions with those used in previous rice studies is presented in Table S1. Garg *et al.*^13^ reported the responses of rice seedlings to six hormones: auxin, CK, SA, ethylene, ABA, and JA. We made a list of predicted orthologs of the *Brachypodium* DEGs in rice and evaluated whether they are regulated in the same manner (Table 6). Since Garg *et al.*^13^ reported phytohormone-regulated DEGs in rice without statistical information for each hormone treatment, we simply checked whether the orthologous genes from *Brachypodium* were included in the list of Garg *et al.*^13^ or not. We predicted 408 orthologs to the *Brachypodium* DEGs in rice. In total, 326 orthologous genes (80%) were regulated by phytohormones in the same manner. The transcriptional response to CK and ABA in *Brachypodium* was consistent with that in rice: 100% of the orthologs of the *Brachypodium* DEGs that were regulated by CK (tZ) were also regulated in rice by CK (BAP), and 98% of the orthologous genes were regulated by ABA in rice. *Brachypodium* and rice shared many auxin- and JA-responsive orthologous genes: 67% of the orthologs were also regulated by IAA in rice. Garg *et al.*^13^ reported that *Aux/IAA* and *GH3* are IAA-responsive gene families. Consistent with this report, many genes in the *Aux/IAA* family (*Bradi1g55370*, *Bradi2g04910*, *Bradi2g34030*, *Bradi2g16850*, *Bradi2g46420*, *Bradi2g31820*, *Bradi3g55410*, *Bradi4g35960*, *Bradi3g54610*, and *Bradi2g11120*) and *GH3* family (*Bradi2g50840*, *Bradi1g22830*, and *Bradi2g52000*) were found to be auxin-responsive in *Brachypodium.* In total, 42% of the orthologous genes were JA-responsive in rice. *JAZ1-like* genes in *Brachypodium* (*Bradi5g24410*), *OsJAZ1*, a *MYC2-like* gene in *Brachypodium* (*Bradi3g34200*), and *OsMYC2* were regulated in both plants. Transcriptional responses to SA and ethylene were less conserved. Only 26% and 31% of the orthologous genes were regulated by SA and ethylene in rice. A transcription factor gene, *OsWRKY45*, which acts in SA-mediated pathogen resistance^17^, and its orthologous gene in *Brachypodium* (*Bradi2g44270*), were found to be regulated by SA in both plants. *Ethylene receptor-like* genes (*Bradi5g00700* and *LOC_Os04g08740*) were found to be regulated in both plants. Sato *et al.*^14^ performed a transcriptional analysis using auxin, CK, GA, BR, ABA, and JA and presented a coexpression database. Since they did not show phytohormone-regulated genes, we did not directly compare our hormone-induced gene expression results with those of Sato *et al.*^14^.

A gene ontology (GO) term enrichment (GOE) analysis was performed to evaluate the DEGs for *Brachypodium*. In this study, the GO terms for *Brachypodium* were redefined using the latest GO annotation for *Arabidopsis thaliana* (see the Methods for details) because the GO terms inferred from InterProScan (http://geneontology.org/page/go-annotation-standard-operating-procedures) lack useful GO terms for the study of phytohormone responses (e.g., the “Response to cytokinin stimulus”; Table S2). Therefore in the following sections, a detailed analysis was performed with the help of GO terms predicted from *Arabidopsis* (Tables 7 and 8). In this study, we analyzed the GOE for *Arabidopsis* using the latest GO terms (R package ath1121501cdf version 2.14.0) and microarray data provided by Goda *et al.*^11^ (Table S3) to compare the results of GOE analyses with those in *Brachypodium*.

### Auxin response

**Figure 1:**
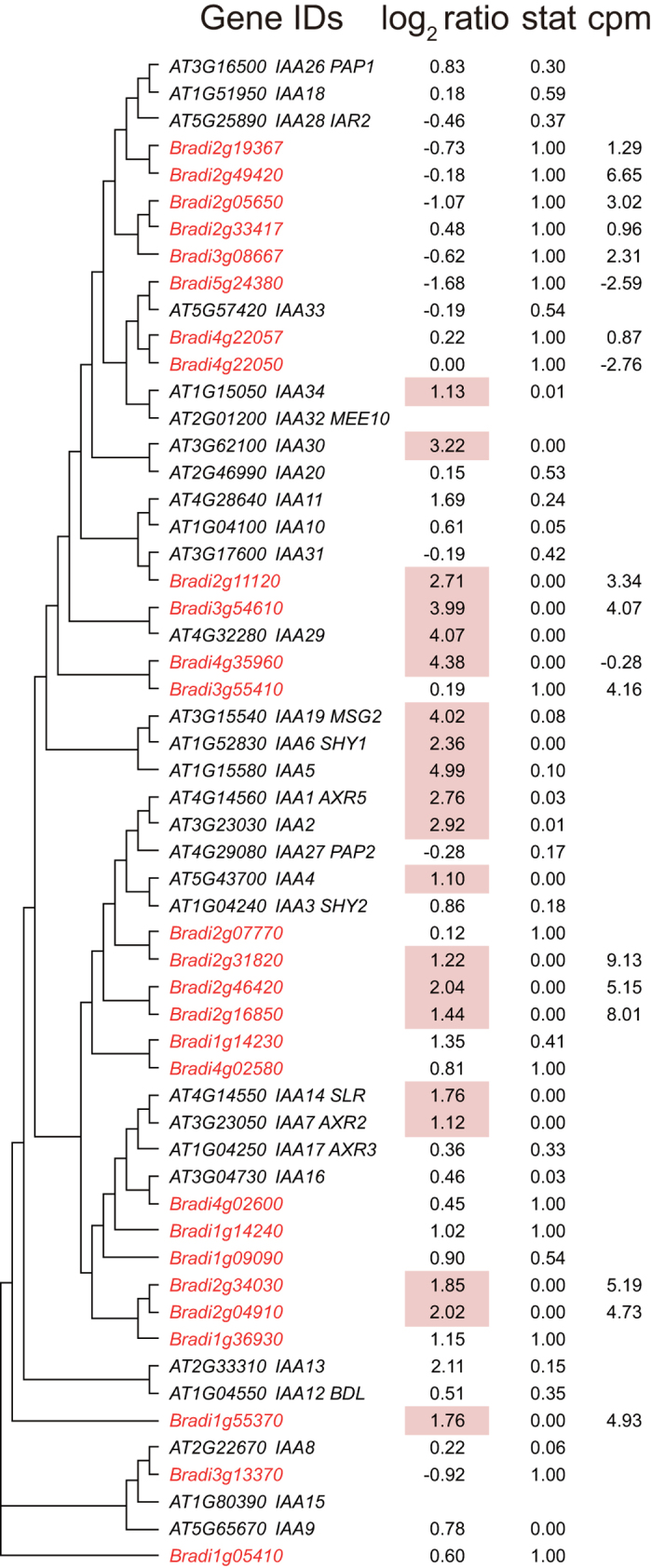
Phylogenic tree of *Aux/IAA* genes and their transcriptional responses to auxin in *Brachypodium* and *Arabidopsis*. *Brachypodium Aux/IAA* family members were retrieved using the BLAST software. Protein sequences were aligned with MAFFT and similarities (percentage identity) are shown as a phylogenetic tree. *Brachypodium* genes are shown in red and *Arabidopsis* genes in black. log_2_ ratio represents the gene expression ratio between the read count in IAA treatment divided by the average read count from all other treatments. Stat represents the p-value of the microarray experiment of *Arabidopsis* treated with IAA^11^ or the FDR of RNA-seq data when the count of IAA treatment was compared with all other treatments. log_2_ ratio is hatched in red if genes are up-regulated. cpm represents the average of log_2_-scaled read counts per million reads in all experiments.

One hundred and twenty-five genes were identified as auxin-responsive genes in the high-stringency analysis (Data S1). The genes with the term “response to auxin stimulus” were found to be enriched in the GOE analysis of auxin-responsive genes in *Brachypodium* (Table 7). As we described in the comparison with rice, many genes in the *Aux/IAA* family and *GH3* family were detected as auxin-responsive genes in *Brachypodium.* These results are also consistent with the auxin response in *Arabidopsis* (Fig. 1, Fig. S1). In contrast to the auxin response in *Arabidopsis*, *Brachypodium* genes related to ethylene functions were not regulated in the same manner (Fig. S2), although their expression levels were not low (present at sufficiently high levels for analysis) (log_2_ cpm (number of counts per million reads) >1).

### CK response

**Figure 2:**
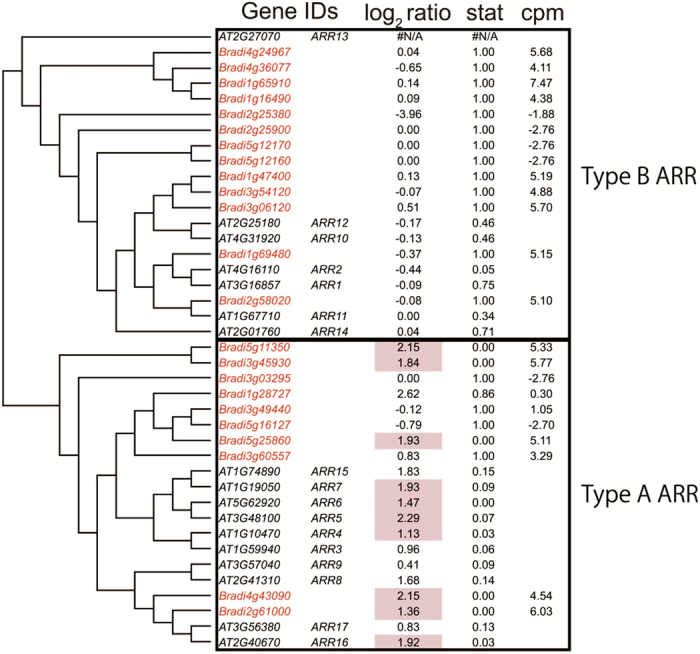
Phylogenic tree of *ARR* genes and their transcriptional responses to CK in *Brachypodium* and *Arabidopsis*. *Brachypodium ARR* family members were retrieved using the BLAST software. Similarities in protein sequences (percentage identity) are shown as a phylogenetic tree. *Brachypodium* genes are shown in red and *Arabidopsis* genes in black. log_2_ ratio represents the gene expression ratio between the read count in tZ treatments divided by the average read count from all other treatments. Stat represents the p-value of the microarray experiment in *Arabidopsis* treated with t-zeatin for 1 h^11^ or FDR of RNA-seq data when the count from tZ treatment was compared with all other treatments. log_2_ ratio is hatched in red if genes are up-regulated. cpm represents the average of log_2_-scaled read counts per million reads from all experiments.

Twenty-three genes were identified as cytokinin-responsive genes in the high-stringency analysis (Data S2). The genes with the term “response to cytokinin stimulus” were enriched in the GOE analysis (Table 7). Genes in the type A *ARR* family (*Bradi2g61000*, *Bradi4g43090*, *Bradi5g25860*, *Bradi3g45930* and *Bradi5g11350*) were detected as cytokinin-responsive genes (Fig. 2). In contrast, members of the type B *ARR* gene family were not detected as cytokinin-responsive genes. These results are consistent with the cytokinin response in *Arabidopsis*^18^.

### SA response

**Figure 3:**
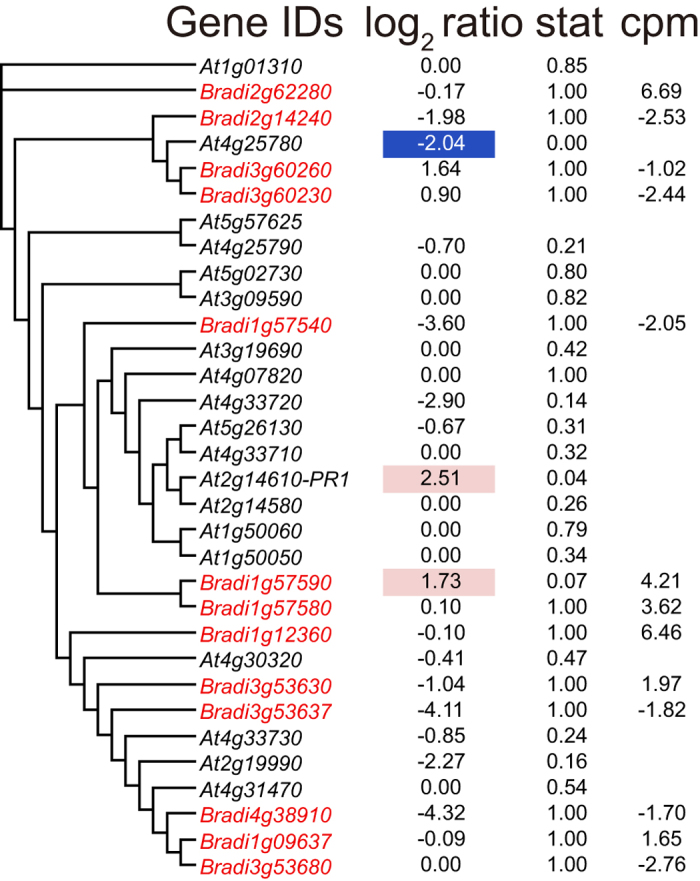
Phylogenic tree of *PR1*, its orthologs and transcriptional responses to SA treatment in *Brachypodium* and *Arabidopsis*. *Brachypodium PR1-*like genes were retrieved using the BLAST software. Similarities in protein sequences (percentage identity) are shown as a phylogenetic tree. *Brachypodium* genes are shown in red and *Arabidopsis* genes in black. log_2_ ratio represents the gene expression ratio between read counts from SA treatment divided by the average read counts from all other treatments. Stat represents the p-value of the microarray experiment. *Arabidopsis* treated with SA for 3 h^11^ for *Arabidopsis* genes or FDR of RNA-seq data when the counts from SA treatment were compared with all other treatments for *Brachypodium* genes. log_2_ ratio is hatched in red if genes are up-regulated and in blue if down-regulated. cpm represents the log_2_-scaled read count per million reads of RNA-seq data from all treatments.

Eighty-one genes were identified as SA-responsive genes in the high-stringency analysis (Data S3). Genes with the term “defense response to fungus” were enriched in GOE analysis (Table 7). A homolog of the *PR-1* gene (*Bradi1g57590*) was up-regulated by SA treatment (Fig. 3).

### ABA response

Four hundred and forty-five genes were identified as ABA-responsive genes in the high-stringency analysis (Data S4). Genes with the term “response to abscisic acid stimulus” were enriched in the GOE analysis (Table 7). Genes homologous to those encoding transcription factors involved in transcriptional regulation in response to ABA (i.e., *ABF*, *NAC*, *Myb*, *bHLH*) were detected as DEGs (*Bradi3g00730*, *Bradi1g59982*, *Bradi3g50220*, *Bradi4g32090*, *Bradi5g17170*, *Bradi2g22660*, *Bradi3g57960*, *Bradi3g19010*, *Bradi3g38200*, *Bradi4g29380*, *Bradi1g22180*, *Bradi1g11800*, *Bradi2g41530*, *Bradi1g20360*, *Bradi4g35910*).

### JA response

**Figure 4:**
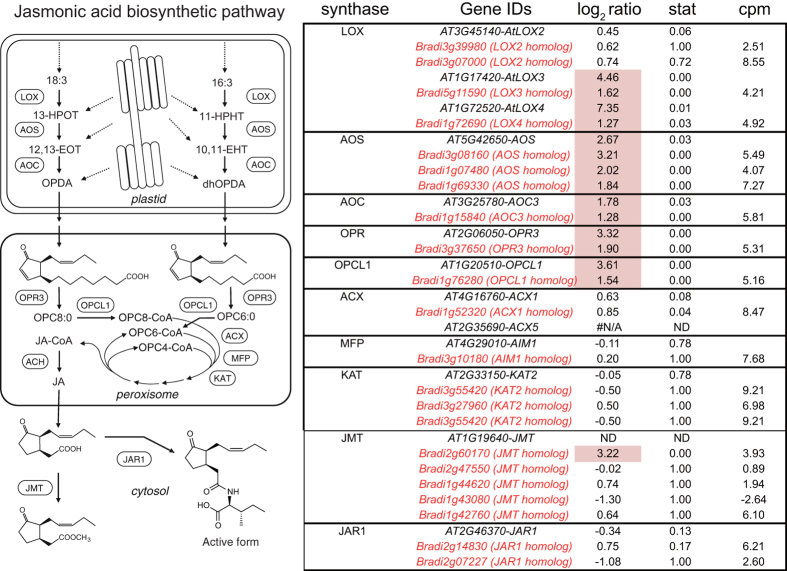
Biosynthesis pathway of JA and transcriptional responses in *Brachypodium* and *Arabidopsis*. Homologs of jasmonate biosynthetic genes in *Brachypodium* were retrieved using the BLAST software. *Brachypodium* genes are shown in red and *Arabidopsis* genes in black. Stat represents the p-value of the microarray experiment. *Arabidopsis* treated by MJ for 3 hours^11^ and FDR of RNA-seq data when the count of MJ treatment was compared with all other treatments. log_2_ ratio represents the gene expression ratio between read counts from the MJ treatment divided by the average read counts from all other treatments. log_2_ ratio is hatched in red if genes are up-regulated. cpm represents log_2_ scaled read count per million reads of RNA-seq data from all treatments.

Three hundred and eighty-three genes were identified as JA-responsive genes in the high-stringency analysis (Data S5). Genes with the term “response to wounding” were enriched in the GOE analysis (Table 7). Genes involved in the biosynthesis of jasmonic acid (*LOX3* homolog (*Bradi5g11590*), *LOX4* homolog (*Bradi1g72690*), *AOS* homolog (*Bradi3g08160*, *Bradi1g07480*, *Bradi1g69330*), *AOC3* homolog (*Bradi1g15840*), *OPR3* homolog (*Bradi3g37650*) and *OPCL1* homolog (*Bradi1g76280*) were up-regulated by MJ treatment (Fig. 4). The *JAZ* genes in *Arabidopsis* encode JA receptors^19^. Two members of the *JAZ* family (*Bradi3g23190*, *Bradi1g58490*) were also up-regulated in *Brachypodium*.

### GA response

No gene was detected as a DEG in the high-stringency analysis (Data S6). This indicates that the GA-inducible gene expression response is relatively weaker than those to other hormones, which is consistent with the GA response in *Arabidopsis*^11^. When the effects on gene expression of the GA biosysthesis inhibitor Phx treatment were compared to those of the mock 3-h and Phx+GA treatments (low-stringency analysis), 224 genes were detected as DEGs (Data S14). Of these genes, those with the term “response to nitrate” were the most enriched in the GOE analysis (Table 8). In *Arabidopsis*, many GA-biosynthesis genes are also GA-responsive, since there is a negative feedback regulation in the expression of GA-biosynthesis genes^11^. The read counts of many genes involved in gibberellin biosynthesis (homologs of *GA2oxs*, *GA3oxs* and *GA20oxs*; i.e., *Bradi1g56200*, *Bradi1g56210*, *Bradi1g56220*, *Bradi2g16727*, *Bradi4g23540*, *Bradi2g16750*, *Bradi3g49390*, *Bradi2g32577*, *Bradi2g24980*, *Bradi5g16040*, *Bradi1g59570*, *Bradi2g06670*, *Bradi2g57027*, *Bradi2g34837*, *Bradi2g19900*, *Bradi2g50280*) were low, and none were detected as DEGs (log_2_ cpm <1, Table S4). Genes of *Xyloglucan endotransglucosylase/hydrolases TCH4* (*Bradi3g18690*) and *XTH12* (*Bradi3g10310*) were significantly up-regulated. Many genes in *Peroxidase superfamily* (*Bradi1g17860, Bradi1g17877, Bradi5g27160, Bradi2g38690, Bradi2g38700, Bradi1g44790, Bradi1g59520, Bradi2g20830, Bradi2g38670, Bradi3g33780, Bradi1g61550, Bradi2g11320, Bradi3g10470, Bradi5g10070, Bradi2g38685, Bradi1g61530, Bradi2g12180, Bradi2g20850, Bradi1g20020, Bradi2g20840, Bradi2g09660, Bradi1g33730, Bradi1g44800, Bradi2g38680, Bradi3g54010*) were also up-regulated.

### BR response

**Figure 5:**
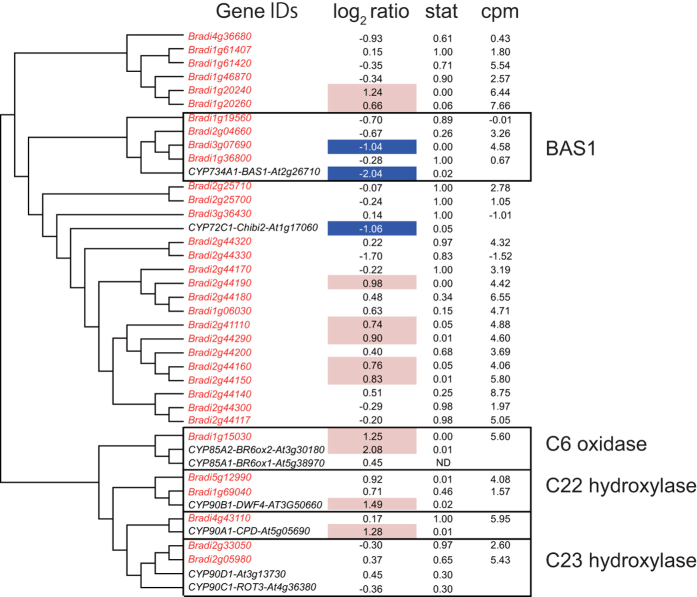
*P450* genes involved in BR biosynthesis and transcriptional responses to Brz in *Brachypodium* and *Arabidopsis*. *Brachypodium* BR biosynthetic genes were retrieved using the BLAST software. Similarities in protein sequences (percentage identity) are shown as a phylogenetic tree. *Brachypodium* genes are shown in red and *Arabidopsis* genes in black. log_2_ ratio represents the gene expression ratio between read counts from the Brz treatment divided by the average read counts from mock 1-h and Brz+BL treatments. Stat represents the p-value of the microarray experiment, *Arabidopsis det2* compared with wild-type^11^ for *Arabidopsis* genes or FDR of RNA-seq data when the counts from the Brz treatment were compared with mock 1-h and Brz+BL treatments for *Brachypodium* genes. log_2_ ratio is hatched in red if genes are up-regulated and in blue if down-regulated. cpm represents log_2_ scaled read counts per million reads of RNA-seq data from mock 1-h, Brz and Brz+BL treatments.

Only four genes were detected as DEGs in the Brz220 treatment compared with all other treatments (high-stringency analysis, Data S7). This indicates that the BR-inducible gene expression response is relatively weak compared with those to other hormones, which is consistent with the BR response in *Arabidopsis*^11^. When the effects on gene expression of the Brz220 treatment were compared with those of the mock 3-h and Brz220+BL treatments (low-stringency analysis), 474 genes were detected as DEGs (Data S15). Of these genes, those with the term “RNA elongation” were enriched in the GOE analysis (Table 8). Genes with the term “photosynthesis” were also enriched in the GOE analysis. P450 genes involved in the biosynthesis of brassinosteroids (*Bradi1g15030*, homolog of *BR6ox2* and *Bradi5g12990*, homolog of *DWF4*) were up-regulated by Brz220 compared to the mock 3-h and Brz220+BL treatments (Fig. 5), although the fold change of *Bradi5g12990* was less than twice. This is consistent with the BR response in *Arabidopsis*; *BR6ox2* and *DWF4* were up-regulated in the *det2* mutant of *Arabidopsis* compared to the wild-type (Fig. 5).

### Ethylene response

No gene was detected as a DEG in response to ethylene in the high-stringency analysis (Data S8). To confirm response of seedlings to ACC treatment under our experimental conditions, plants were treated with ACC for a further week. Seedlings showed growth inhibition when grown in the presence of 10 μM ACC (Fig. S3), indicating that plants respond to exogenous ACC. Therefore, gene expression in response to ACC treatment was compared to that in response to the mock 1-h and mock 3-h treatments (low-stringency analysis). Fifty-two genes were detected as DEGs (Data S16). Of these DEGs, those with the term “RNA elongation” were enriched in GOE analysis (Table 8). A gene homologous to *EIN4*, which is involved in sensing ethylene (*Bradi5g00700*) was up-regulated, and a gene homologous to *ACS6*, which is a member of the ethylene synthesis gene family, (*Bradi5g19100*) was down-regulated by ethylene treatment compared to the mock 1-h and mock 3-h treatments.

## Discussion

The comprehensive transcriptional response of *Brachypodium* to phytohormones was analyzed in this study. To obtain clear responses from *Brachypodium*, we optimized the experimental design using the expressions of selected *Brachypodium* marker genes to validate hormone response. The concentrations of phytohormones used in this study were similar (1–10-fold) to those used in the analysis of the response to hormones in rice and *Arabidopsis* (Table S1), with the exception of the concentration of BL (100-fold compared to that used for *Arabidopsis*). We successfully identified BR- and GA-regulated genes (e.g., *Bradi1g15030*, homolog of *BR6ox2* as a BR-responsive gene and *Bradi3g18690,* homolog of *TCH4* as a GA-responsive gene).

In rice, 80% of the orthologs of the *Brachypodium* DEGs were also regulated by CK, ABA, auxin, JA, SA, and ethylene. These data show that major transcriptional responses to these phytohormones are shared in monocots, although fewer orthologous genes were commonly regulated by SA and ethylene. It was reported that the SA level in rice was the highest among the tested plants^20^. This may be one reason for the different responses to SA at similar concentrations. The difference in ethylene-regulated genes between rice and *Brachypodium* was also reported by Pacheco-Villalobos *et al.*^21^. Sato *et al.*^14^ also reported the responses of rice seedlings to six hormones: auxin, CK, GA, BR, ABA, and JA. However, it was difficult to compare our data with theirs because they reported their data as part of a large transcriptome dataset and no analysis of hormone-responsive gene expression was performed using the data.

The transcriptional responses to phytohormones and the mechanisms involved in transcriptional regulation in *Arabidopsis* are well characterized. In addition, manually curated GO terms for Arabidopsis is very helpful to analyze large transcriptome dataset. Therefore, we compared the transcriptional response to phytohormones in *Brachypodium* to that in *Arabidopsis thaliana* in detail. A comprehensive analysis of phytohormone-regulated genes of *Brachypodium* revealed common transcriptional responses between *Brachypodium* and *Arabidopsis*. To compare the accumulation of GO terms in *Brachypodium* to *Arabidopsis*, we assigned new GO terms to the *Brachypodium* genes using the *Arabidopsis* GO terms. Some results of the GOE analyses in *Arabidopsis* and *Brachypodium* were common following treatment with the same phytohormones. The GO terms “response to auxin stimulus” had the lowest p-values in auxin-regulated DEGs. Similarly, “cellular response to cytokinin stimulus” in CK-regulated DEGs, “defense response to fungus” in SA-regulated DEGs, “response to abscisic acid stimulus” in ABA-regulated DEGs and “response to wounding” in JA-regulated DEGs had the lowest p-values in *Brachypodium* (Tables 7, 8). These GO terms were also enriched in the hormone-responsive genes in *Arabidopsis* (Table S3). The GO term “response to nitrate” was enriched in DEGs in the Phx treatment (Table 7), which was consistent with the enriched GO of the GA-biosynthesis mutant *ga1-5* when compared to the wild type (Table S5). The GO term “photosynthesis” was enriched in DEGs of Brz-treated *Brachypodium* (Table 7), which was consistent with the enriched GO terms of the BR-biosynthesis mutant *det2* when compared to wild-type *Arabidopsis*. Consistent with the findings in BR-treated *Arabidopsis det2*, genes involved in BR-synthesis were detected as DEGs in Brz-treated *Brachypodium* (Fig. 5). Fewer were detected as DEGs in response to ACC than to other hormones (Tables 4, 5). This was also consistent with transcriptional regulation in ethylene-treated *Arabidopsis*^11^,^15^. These results suggest that many molecular mechanisms characteristic of the responses to phytohormones in *Arabidopsis* are also present in *Brachypodium*.

In contrast, there were some differences in the hormone responses of *Brachypodium* and *Arabidopsis*. In *Arabidopsis*, genes involved in ethylene synthesis and signaling, *ACO1*, *ACO3*, *ACO4/EFE*, *ACS4*, *ACS8*, and *ERF1*, are responsive to auxin treatment. Only one gene from the *ACO* gene family (*Bradi2g41840*) was detected as a DEG in auxin-treated *Brachypodium* (Fig. S2). Genes involved in ethylene signaling, such as *ERF2*, *8*, *11*, *ERS1*, *EBF2*, *ETR2*, *CTR1* and *EIN2*, are regulated by ACC treatment in *Arabidopsis*^15^. However, in *Brachypodium*, only one gene homologous to *EIN4* (*Bradi5g00700*) was detected as a DEG. These differences in auxin- and ethylene-responsive genes suggest divergent auxin-ethylene relationships between eudicots and monocots. Pacheco-Villalobos *et al.*^21^ reported that the cross-talk between ethylene and auxin in *Brachypodium* differs from that in *Arabidopsis*. Further analysis of these differentially expressed genes will improve our understanding of the different functions of these hormones and the related genes in *Brachypodium* and *Arabidopsis*.

We encountered some difficulties in the analysis of RNA-seq data. We prepared 13–30 million reads because Liu *et al.*^22^ reported that the increase in number of DEGs drops after 10–15 million reads. However, the transcriptional responses of some genes might not have been detected because of their low read counts. For example, none of the genes involved in GA-biosynthesis was detected as Phx- or GA-responsive in *Brachypodium*. However, it was reported that the expressions of the GA-biosynthetic genes *AtGA2ox1*, *AtGA2ox2*, *GA3ox1* and *GA20ox1* changed in *Arabidopsis* GA-deficient mutants following endogenous GA treatment^23^,^24^,^25^,^26^. The read counts of these GA-biosynthetic genes in *Brachypodium* were low (Table S4). In *Arabidopsis* and rice, few genes respond to ethylene^11^,^13^. In this study, no gene in the expansin family was detected as ethylene-responsive in *Brachypodium*, while *AtEXP8* and *AtEXP18* in *Arabidopsis*^27^ and *OsEXP1, OsEXP2* in rice^13^ are responsive. The expression levels of 27 expansin genes were low (cpm <1, Table S6). The growth of *Brachypodium* was reduced by ACC treatment (Fig. S3); therefore, some expansin genes may be regulated by ethylene to control cell elongation. To examine the transcriptional regulation of these poorly expressed genes, further analysis using additional RNA-seq reads with different tissues or different growth stages, and/or complementary methods (such as microarray or quantitative PCR) are required.

## Methods

### Plant materials

Seedlings of *Brachypodium distachyon* Bd21 were grown on 1/2 MS medium^28^ supplemented with 1% sucrose and 0.8% agar 4 days after breaking dormancy at 4 °C in the dark. The plants were grown on agar plates for 4 days and transplanted to 1/2 MS liquid culture with 1% sucrose. The plants were pre-incubated for 24 h in liquid culture with shaking (70–80 rpm) and then treated with phytohormones (Table 2). The phytohormones were dissolved in DMSO before addition to the liquid cultures. DMSO (0.1% (v/v)) was used for mock treatments. For the “Inhibitor and GA” and “Inhibitor and BR” treatments, GA and BL were added to liquid cultures 2 h after treatment with the GA inhibitor, prohexadione-calcium (Wako), or BR-inhibitor, brassinazole220^29^, respectively. All growth and treatment processes were performed at 22 °C under continuous light. Phytohormone treatments were conducted twice independently as biological replicates, and the hormone responses were confirmed using the transcriptional responses of marker genes (Table 1). Transcriptional responses of marker genes were checked with RT-PCR using gene specific primers (Table S7) prior to the RNA-seq analysis.

### RNA extraction

RNA was extracted from each sample using the RNeasy Mini Kit (QIAGEN). The concentration and quality of the RNA samples were determined using a Nanodrop 2000 instrument (Thermo Scientific). RNA extracted from two biological replicates was mixed and used for the RNA-seq analysis.

### Strand-specific RNA-seq analysis

Strand-specific RNA libraries were prepared using the TruSeq Small RNA Sample Prep Kit (Illumina) and TruSeq RNA sample Preparation Kit v2 (Illumina) following the instructions in the Directional mRNA-seq Library Prep. (Pre-Release Protocol Rev.A (Illumina)). Fragmented Poly(A)-RNA was treated with T4 polynucleotide kinase (TAKARA) for phosphorylation of the 5′ end. An RNA 3′ adopter was ligated to the 3′ end using T4 RNA ligase 2, truncated (NEB), then an RNA 5′ adopter was ligated to the 5′ end using T4 RNA ligase 1 (NEB). Single-strand cDNA was synthesized with a primer for the RNA 3′ adaptor. Amplified cDNA was size-selected using 6% polyacrylamide gel electrophoresis, then used as the sequencing library. The quality of all libraries was assessed using a Bioanalyzer 2100 (Agilent). Sequencing was performed using a HiSeq2000 sequencer (Illumina) to sequence 100 bp. The sequencing was performed with the single-read method. The sequence data have been submitted to the DDBJ Sequence Read Archive under accession number PRJDB2997.

### Mapping of RNA-Seq reads, transcript assembly and abundance estimation

The sequences were aligned to the Phytozome 9.0 *Brachypodium distachyon* reference genome Bdistachyon_192 (Bdistachyon_192_hardmasked.fa.gz) using TopHat v2.0.11^30^, which is integrated with Bowtie v2.2.1^31^. The Bowtie index was generated from Bdistachyon_192 and counting of the mapped reads was performed using Cufflinks v2.2.1^32^ and genome annotation reference PASA_updates.phytozome.gff3^4^. Read counts were generated using Cuffdiff 32. Differentially expressed genes (DEGs) were identified with edgeR^16^, as follows. Parameters and function usages in edgeR were determined to maximize the specificity for detecting hormone marker genes (Table 1) as DEGs. Read counts were normalized with the function “calcNormFactors”. The TMM normalization method in edgeR can normalize different amounts of RNA-Seq data without increasing the false-positive rate of detecting DEGs^33^. Dispersions among genes and among replications were estimated by the functions “estimateGLMCommonDisp”, “estimateGLMTrendedDisp” and “estimateGLMTagwiseDisp” and p-values were calculated using the likelihood-ratio test with the generalized linear models. Calculated p-values were adjusted using the false discovery rate (FDR) of Benjamini and Hochberg’s approach^34^. DEGs were defined as FDR <0.05 and up- or down-regulated genes were identified in the DEGs as those having a log_2_ ratio of cpm >1 or that of cpm <−1, respectively.

### Phytohormone-regulated genes in *Arabidopsis*

Microarray data from *Arabidopsis thaliana* treated with phytohormones were retrieved from the AtGenExpress project^11^. Signals in the microarray data were calculated using R package affy (1.42.3) with the MAS5 method^35^. DEGs were identified as p-value < 0.1 by Student’s *t*-test and up- and down-regulated genes were defined in the DEGs as log_2_ ratios of signals >1 and <−1, respectively. GOE analyses were performed with the DEGs.

### Determination of orthologous/homologous genes and generation of the phylogenetic tree

To obtain orthologous gene pairs, *Arabidopsis* protein sequences (TAIR10) and rice protein sequences (RGAP 7) were applied to BLAST (blastp) to search for homologous sequences in the *Brachypodium* protein sequence (Bdistachyon_192_peptide.fa from Phytozome 9.0). *Brachypodium* sequences with top hit were collected and applied to BLAST again to search for the most similar sequences in *Arabidopsis* and rice. If the results of the second BLAST returned the former Arabidopsis or rice sequence, the corresponding gene pairs were selected as orthologous gene pairs. To obtain genes from each gene family in *Brachypodium*, protein sequences of members of each family in *Arabidopsis* were applied to BLAST (blastp with default settings) to search for similar sequences in *Brachypodium* (Bdistachyon_192_peptide.fa). *Brachypodium* sequences with E-values less than thresholds were collected and the corresponding *Brachypodium* genes were selected as family members (Figs 1, 2, 3, 4, 5 and Figs S1,S2). To obtain superfamily or functionally related subfamily, optimal E-value for each gene family was selected. For the *Aux/IAA* gene family, 19 protein sequences of *Arabidopsis* Aux/IAA1-19 with a threshold E-value < 10^−10^ were used. For the *ARR* family, 17 protein sequences of *Arabidopsis* ARR1-17 with a threshold E-value < 10^−5^ were used. For the *GH3* gene family, sequences of *Arabidopsis* genes in Fig. S1 with a threshold E-value < 0.1 were used. For the *ACS* and *ACO* gene families, 11 protein sequences (ACS1-11) and 4 proteins (ACO1-4) with a threshold E-value < 10^−10^ were used. To obtain homologous BR-biosynthetic *P450* genes from *Brachypodium*, the protein sequences of BAS1, Chibi2, BR6ox2, BR6ox1, DWF4, CPD, ROT3 and CYP90D1/At3g13730 were applied to BLAST (blastp) to search for similar sequences in the *Brachypodium* protein sequence. *Brachypodium* sequences with an E-value < 10^−100^ were collected and the corresponding *Brachypodium* genes were selected as BR-biosynthetic genes. To identify SA-responsive genes homologous to *PR1* in *Brachypodium*, the protein sequence of PR1 and its *Arabidopsis* homologous proteins (BLAST E-value < 10^−10^) were applied to BLAST (blastp) to search for similar sequences in the *Brachypodium* protein sequence. *Brachypodium* sequences with an E-value < 10^−10^ were collected and the corresponding *Brachypodium* genes were selected as *PR1-like* genes. Similarly, to identify genes homologous to *ERF1*, the ERF1 protein sequence was applied to BLAST and those with an E-value < 10^−10^ were selected as *ERF1-like* genes. To identify genes homologous to JA-biosynthetic genes in *Brachypodium*, the protein sequences of AtLOX2-4, AOS, AOC3, OPR3, OPCL1, ACX1, ACX5, AIM1, KAT2, JMT and JAR were applied to BLAST (blastp) to search for homologous sequences in the *Brachypodium* protein sequence. *Brachypodium* sequences with an E-value < 10^−10^ were collected and applied to BLAST again to search for the most similar sequences in *Arabidopsis*. If the results of the second BLAST returned AtLOX2-4, AOS, AOC3, OPR3, OPCL1, ACX1, ACX5, AIM1, KAT2, JMT or JAR, the corresponding *Brachypodium* genes were selected as being homologous JA-biosynthetic genes. To create the phylogenetic tree of orthologous genes in *Brachypodium* and *Arabidopsis*, translated amino acid sequences were aligned with MAFFT^36^ using the “auto” settings. The neighbor-joining tree^37^ of the aligned sequences was then generated using percent identity and visualized as a Rectangular Cladogram in Dendroscope^38^.

### Gene ontology term enrichment (GOE) analysis

GOE analysis was carried out using the following procedure. First, *Brachypodium* protein sequences were applied to BLAST (tblastn) to search for the most similar gene in *Arabidopsis thaliana*. GO terms of the top hits in *Arabidopsis* were copied as the customized annotations of corresponding *Brachypodium* genes. GOE analysis was performed using AgriGO^39^ with customized annotations as the query, customized annotated reference and with default settings for statistical analysis (p-value was calculated with Fisher’s exact test and “FDR with Yekutieli”).

## Additional Information

**How to cite this article**: Kakei, Y. *et al.* Transcriptome analysis of hormone-induced gene expression in *Brachypodium distachyon.* *Sci. Rep.* **5**, 14476; doi: 10.1038/srep14476 (2015).

## Supplementary Material

Supplementary Information

Supplementary Dataset

## Figures and Tables

**Table 1:** Selection of marker genes.

**Hormone**	**Arabidopsis (Rice) gene**	***Brachypodium* gene**	***Brachypodium* qPCR**
Auxin	*Aux/IAA16*	*Bradi4g02600.1*	No response
Auxin	*Aux/IAA9*	*Bradi2g31820.1*	Responded
Auxin	*Aux/IAA18*	*Bradi2g33417.1*	Responded
Auxin	*(LOC_Os02g57250)*	*Bradi3g55410.1*	Responded
CK	*AtARR1*	*Bradi1g69480.1*	No response
CK	*AtARR9*	*Bradi2g61000.1*	Responded
CK	*(OsRR10)*	*Bradi4g43090.2*	Responded
BR	*BR6ox2*	*Bradi1g15030.1*	Responded
BR	*dwf4*	*Bradi1g69040.1*	Responded
BR	*CPD*	*Bradi4g43110.1*	No response
GA	*GAI*	*Bradi1g11090.1*	Responded
GA	*SCL3*	*Bradi2g60750.1*	No response
GA	*AtEXP1*	*Bradi2g22290.1*	Responded
ABA	*AT1G79520*	*Bradi2g02050.1*	No response
ABA	*HsfA6B*	*Bradi3g26920.1*	Responded
ABA	*GLTP*	*Bradi1g11280.1*	Responded
ABA	*(LOC_Os09g21120)*	*Bradi4g29360.1*	Responded
Ethylene	*ERS2*	*Bradi3g55730.1*	No response
Ethylene	*EFE,ACO4*	*Bradi3g57620.1*	No response
Ethylene	*DL4170C*	*Bradi2g27140.1*	No response
Ethylene	*(OsETR2)*	*Bradi5g00700.1*	Responded
Ethylene	*(LOC_Os01g73200)*	*Bradi4g27680.1*	Responded
JA	*JMT*	*Bradi2g47550.1*	No response
JA	*OPR3*	*Bradi3g37650.1*	Responded
JA	*(OsMYC2)*	*Bradi3g34200.1*	Responded
SA	*PR-1*	*Bradi1g57590.1*	Responded
SA	*WRKY70*	*Bradi2g44270.1*	Responded
SA	*AtMES1*	*Bradi2g52110.1*	Responded

**Table 2:** Hormone treatment conditions in *Brachypodium.*

**Hormone treatment**	**Chemicals**	**Concentration**	**Time**
Mock	—	—	3 h
Auxin	IAA	10 μM	3 h
Cytokinin	tZ	1 μM	3 h
Gibberellin inhibitor	Phx	100 μM	5 h
Inhibitor and Gibberellin	Phx+GA4	100 μM+3 μM	2 + 3 h
Brassinosteroid inhibitor	Brz220	100 μM	5 h
Inhibitor and Brassinosteroid	Brz220+BL	100 μM + 1 μM	2 + 3 h
Salicylic acid	SA	100 μM	3 h
Mock	—	—	1 h
Abscisic acid	ABA	10 μM	1 h
Jasmonate	MJ	30 μM	1 h
Ethylene	ACC	100 μM	1 h

**Table 3:** Mapped sequence reads from RNA-seq analysis.

**Treatment**	**No. of reads**	**TopHat2**	
		**No. of mapped reads**	**% of mapped reads**
Mock 3 h	14,564,558	12,987,675	0.89
IAA	13,305,739	12,020,000	0.90
tZ	13,370,016	11,832,703	0.89
Phx	14,504,534	13,124,973	0.90
Phx+GA4	13,140,608	10,548,366	0.80
Brz220	13,651,634	11,005,887	0.81
Brz220+BL	14,590,592	12,002,938	0.82
SA	14,877,403	12,247,895	0.82
Mock 1 h	27,388,326	25,423,449	0.93
ABA	26,462,899	24,717,631	0.93
MJ	29,605,491	27,494,719	0.93
ACC	28,057,412	26,336,415	0.94

**Table 4:** Number of DEGs detected in comparison with all other experiments (high stringency).

**Treatment**	**DEGs**	**Treatment**	**DEGs**
Mock 3 h	0	Mock 1 h	0
IAA	125	ABA	445
tZ	23	MJ	383
Phx	0	ACC	0
Phx+GA4	0		
Brz220	4		
Brz220+BL	0		
SA	81		

**Table 5:** Number of DEGs detected in comparison with two control experiments (low stringency).

**Treatment**	**Control 1**	**Control 2**	**DEGs**
Phx	Mock 3 h	Prohexadion+GA3	224
Brz220	Mock 3 h	Brz220+BL	474
ACC	Mock 1 h	Mock 3 h	52
Others	Mock 1 h	Mock 3 h	–

**Table 6:** Comparison of transcriptional response using orthologous genes of *Brachypodium* and rice.

**Hormone treatment**	**DEGs conserved in rice (%)**
CK	100
ABA	98
IAA	67
JA	42
SA	26
Ethylene	31
Total	80

**Table 7:** GOE analysis of DEGs obtained under high-stringency conditions.

**GOE in Auxin**	**Term**	**P-value**	**FDR**
GO:0009733	Response to auxin stimulus	1.00E-19	1.00E-16
GO:0010311	Lateral root formation	9.90E-16	5.10E-13
GO:0010102	Lateral root morphogenesis	1.20E-13	3.10E-11
GO:0010101	Post-embryonic root morphogenesis	1.20E-13	3.10E-11
GO:0009741	Response to brassinosteroid stimulus	2.80E-12	4.90E-10
GOE in CK			
GO:0071368	Cellular response to cytokinin stimulus	8.60E-07	0.00015
GO:0009736	Cytokinin-mediated signaling pathway	8.10E-07	0.00015
GO:0009735	Response to cytokinin stimulus	4.40E-07	0.00015
GO:0048511	Rhythmic process	5.90E-05	0.0062
GO:0007623	Circadian rhythm	5.90E-05	0.0062
GOE in SA			
GO:0050832	Defense response to fungus	0.0075	0.86
GO:0002252	Immune effector process	0.007	0.86
GO:0006952	Defense response	0.0066	0.86
GO:0006950	Response to stress	0.006	0.86
GO:0010200	Response to chitin	0.006	0.86
GOE in ABA			
GO:0009737	Response to abscisic acid stimulus	4.00E-16	1.00E-12
GO:0009414	Response to water deprivation	7.50E-13	7.90E-10
GO:0009415	Response to water	9.20E-13	7.90E-10
GO:0009725	Response to hormone stimulus	1.50E-08	1.00E-05
GO:0042538	Hyperosmotic salinity response	2.10E-07	0.00011
GOE in JA			
GO:0009611	Response to wounding	7.70E-31	2.50E-27
GO:0009695	Jasmonic acid biosynthetic process	4.20E-23	6.70E-20
GO:0031408	Oxylipin biosynthetic process	6.20E-23	6.70E-20
GO:0009694	Jasmonic acid metabolic process	3.00E-22	2.40E-19
GO:0031407	Oxylipin metabolic process	4.20E-22	2.70E-19

**Table 8:** GOE analysis of DEGs obtained under low-stringency conditions.

**GOE in Phx**	**Term**	**P-value**	**FDR**
GO:0010167	Response to nitrate	3.70E-15	6.20E-12
GO:0015706	Nitrate transport	1.20E-14	1.00E-11
GO:0015698	Inorganic anion transport	5.10E-13	2.80E-10
GO:0010106	Cellular response to iron ion starvation	1.30E-12	5.30E-10
GO:0006826	Iron ion transport	3.30E-11	1.10E-08
GOE in Brz			
GO:0006354	RNA elongation	1.50E-46	4.90E-43
GO:0015979	Photosynthesis	1.10E-23	1.80E-20
GO:0006091	Generation of precursor metabolites and energy	2.00E-17	2.10E-14
GO:0019684	Photosynthesis, light reaction	8.90E-13	7.20E-10
GO:0009145	Purine nucleoside triphosphate biosynthetic process	6.10E-11	3.90E-08
GOE in Ethylene			
GO:0032774	RNA biosynthetic process	0.0011	0.066
GO:0023034	Intracellular signaling pathway	0.00077	0.066
GO:0006350	Transcription	0.0011	0.066
GO:0006351	Transcription, DNA-dependent	0.0011	0.066
GO:0048523	Negative regulation of cellular process	0.00062	0.066
