# Supplementary material for: Transcriptome analysis of hormone-induced gene expression in Brachypodium distachyon
Source: Sci Rep. 2015 Sep 30;5:14476. doi: 10.1038/srep14476 (PMC4588574; doi:10.1038/srep14476)
Supplement: Supplementary Information [file srep14476-s1.pdf]

Transcriptome analysis of hormone-induced gene expression in *Brachypodium distachyon*

Yusuke Kakei, Keiichi Mochida, Tetsuya Sakurai, Takuhiro Yoshida, Kazuo Shinozaki & Yukihisa Shimada

Supplementary information

Figure S1. Phylogenic tree of *GH3* genes and transcriptional responses to auxin in *Brachypodium* and Arabidopsis.

Figure S2. Phylogenic tree of the ethylene-related genes *ACS*, *ACO* and *ERF* and transcriptional responses to auxin in *Brachypodium* and Arabidopsis.

Figure S3. Images of ethylene-treated *Brachyodium* seedlings

Table S1 Comparison of treatments in *Brachypodium*, Arabidopsis and rice

Table S2 GOE analysis of phytohormone-regulated genes with GO terms inferred from InterProScan

Table S3 GOE analysis of phytohormone-regulated genes in Arabidopsis

Table S4 Expression of GA-synthetic genes in *Brachypodium*

Table S5 GOE analysis of *ga1-5* mutant vs Col-0

Table S6 Expressions of expansins

Table S7 Primers for RT-PCR

Supplementary Dataset (SupplementaryDataset.xls)

Data S1 Transcriptional changes by auxin treatment (High stringency analysis)

Data S2 Transcriptional changes by CK treatment (High stringency analysis)

Data S3 Transcriptional changes by SA treatment (High stringency analysis)

Data S4 Transcriptional changes by ABA treatment (High stringency analysis)

Data S5 Transcriptional changes by JA treatment (High stringency analysis)

Data S6 Transcriptional changes by Phx treatment (High stringency analysis)

Data S7 Transcriptional changes by Brz220 treatment (High stringency analysis)

Data S8 Transcriptional changes by Ethylene treatment (High stringency analysis)

Data S9 Transcriptional changes by auxin treatment (Low stringency analysis)

Data S10 Transcriptional changes by CK treatment (Low stringency analysis)

Data S11 Transcriptional changes by SA treatment (Low stringency analysis)

Data S12 Transcriptional changes by ABA treatment (Low stringency analysis)

Data S13 Transcriptional changes by JA treatment (Low stringency analysis)

Data S14 Transcriptional changes by Phx treatment (Low stringency analysis)

Data S15 Transcriptional changes by Brz220 treatment (Low stringency analysis)

Data S16 Transcriptional changes by Ethylene treatment (Low stringency analysis)

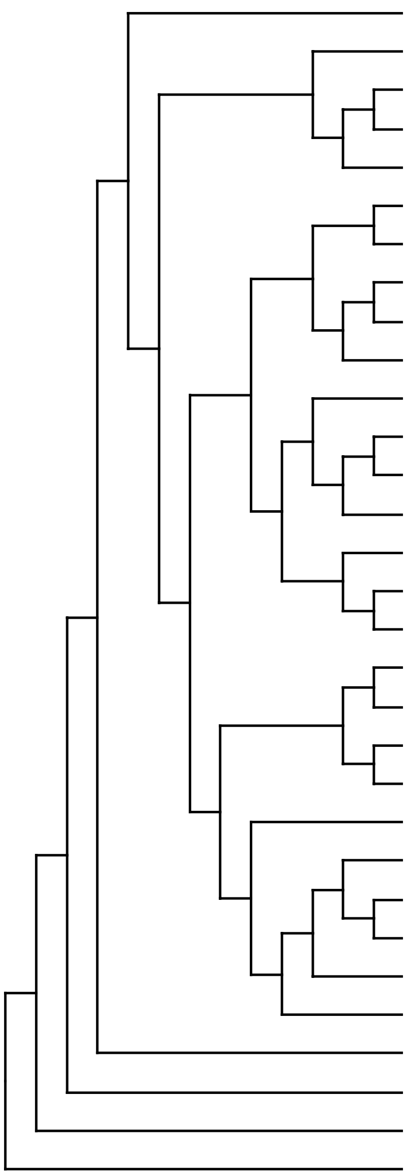

Gene IDs	log_2 ratio	stat	cpm
Bradi1g23840	-0.24	1.00	
At5g13350	0.00	0.72	
At5g13360			
At5g13370			
At5g13380	0.00	0.39	
At5g13320-GH3.12	0.00	0.73	
At1g23160	0.00	0.97	
At1g48660	-2.36	0.28	
At1g48670	0.00	0.26	
At5g51470	0.00	0.17	
At4g03400-GH3.10-DFL2	-0.35	0.54	
Bradi2g14830	-0.47	1.00	6.21
At2g46370-GH3.11	-0.18	0.45	
Bradi2g07227	0.03	1.00	2.60
Bradi1g65610	-0.31	1.00	1.96
Bradi3g12627	0.68	0.82	4.66
Bradi1g09300	0.07	1.00	10.26
At4g27260-GH3.5-WES1	3.70	0.08	
At5g54510-GH3.6-DFL1	2.59	0.06	
Bradi2g52000	2.38	0.00	3.40
Bradi2g20740	0.45	1.00	3.42
Bradi1g22830	6.23	0.00	5.82
At2g23170-GH3.3	5.53	0.05	
At1g59500-GH3.4			
At4g37390-GH3.2-TDK1			
At2g14960-GH3.1	3.64	0.12	
Bradi2g50840	5.33	0.00	4.76
At1g28130-GH3.17	-0.35	0.16	
Bradi4g17237	2.16	0.21	0.65
Bradi1g18200	0.40	1.00	1.11
At2g47750-GH3.9		0.00	

Figure S1. Phylogenic tree of GH3 genes and transcriptional responses to auxin in Brachypodium and Arabidopsis.

GH3 family genes in Brachypodium were retrieved using the BLAST software. Brachypodium genes are shown in red and Arabidopsis genes in black. log2 ratio represents the gene expression ratio between read counts from the IAA treatment divided by average read counts from all other treatments. Stat represents the p-value of the microarray experiment of Arabidopsis treated with IAA (Goda et al., 2008) and FDR of RNA-seq data when the counts from the IAA treatment were compared with all other treatments. log2 ratio is hatched in red if genes were defined as up-regulated. cpm represents log2-scaled read counts per million reads of RNA-seq data from all experiments.

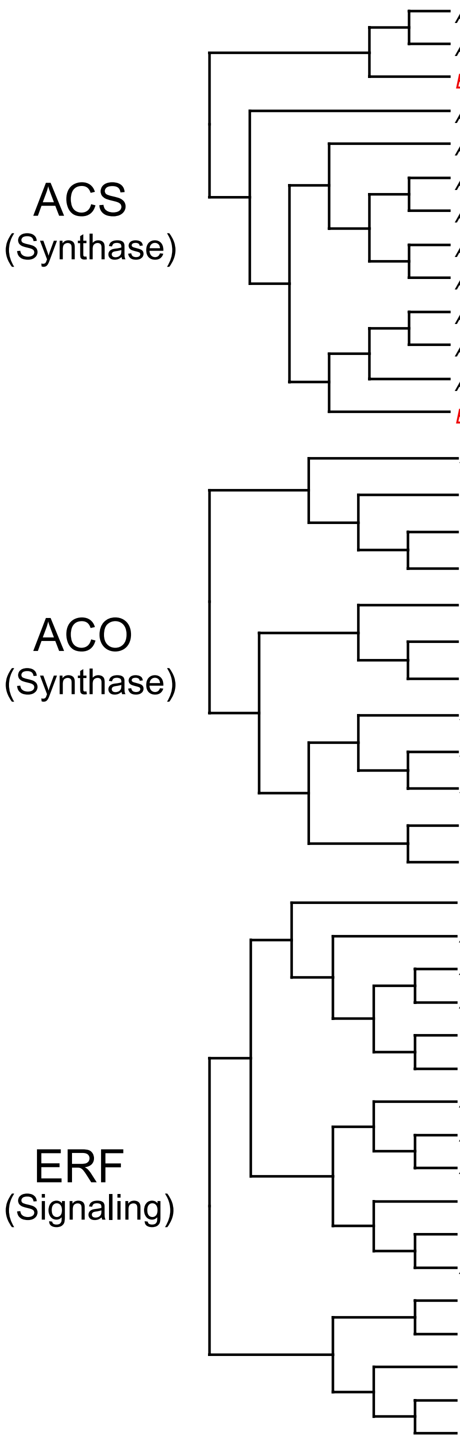

Gene IDs	log_2 ratio	stat	cpm
At1g62960/ACS10	-0.43	0.04	
At5g51690/ACS12	0.16	0.77	
Bradi1g49966	0.15	1.00	5.41
At4g26200/ACS7	0.43	0.12	
At4g08040/ACS11	3.05	0.40	
At2g22810/ACS4	2.31	0.09	
At4g37770/ACS8	3.18	0.00	
At3g49700/ACS9/ETO3	0.00	0.62	
At5g65800/ACS5	0.00	0.87	
At3g61510/ACS1	not available	not available	
At1g01480/ACC2	0.00	0.66	
At4g11280/ACS6	1.61	0.28	
Bradi5g19100	0.34	1.00	4.38
At2g19590-ACO1	-0.36	0.47	
Bradi1g37420	1.39	1.00	-1.36
Bradi2g41840	4.18	0.00	-0.25
Bradi1g33340	0.05	1.00	5.64
Bradi4g23500	0.55	1.00	1.31
Bradi2g35860	-0.92	1.00	4.83
Bradi2g35850	-1.09	1.00	6.33
At1g05010-ACO4-EFE	0.54	0.00	
At1g62380-ACO2	0.34	0.15	
At1g12010-ACO3	-1.25	0.00	
Bradi4g31820	0.37	1.00	6.48
Bradi3g57620	0.41	1.00	2.62
Bradi3g12565	-0.80	1.00	-2.70
At3g23220-ESE1	0.00	0.21	
At1g04370-ERF14			
At5g43410	0.00	0.11	
Bradi3g27690	0.00	1.00	-2.76
Bradi1g75040	0.00	1.00	-2.76
At3g23240-AtERF1	1.87	0.00	
At1g06160-ORA59	0.21	0.73	
At2g31230-EF15	0.00	0.25	
Bradi1g00670	0.22	1.00	0.67
Bradi4g38930	1.02	1.00	-1.75
At4g18450	0.00	0.03	
Bradi3g58015	-4.62	1.00	-1.53
Bradi3g38140	0.08	1.00	-2.21
Bradi2g31480	0.49	1.00	3.44
Bradi2g45530	0.16	1.00	0.53
Bradi2g07357	0.68	1.00	1.37

Figure S2. Phylogenic tree of the ethylene-related genes ACS, ACO and ERF and transcriptional responses to auxin in Brachypodium and Arabidopsis.

ACS, ACO family genes and ERF1-like genes in Brachypodium were retrieved using the BLAST software. Brachypodium genes are shown in red and Arabidopsis genes in black. log2 ratio represents the gene expression ratio between read counts from the IAA treatment divided by the average read counts from all other treatments. Stat represents the p-value from the microarray experiment in Arabidopsis treated with IAA (Goda et al., 2008) and FDR of RNA-seq data when the counts from IAA treatment were compared with all other treatments. log2 ratio is hatched in red if genes are up-regulated. cpm represents log2 scaled read counts per million reads of RNA-seq data from all experiments.

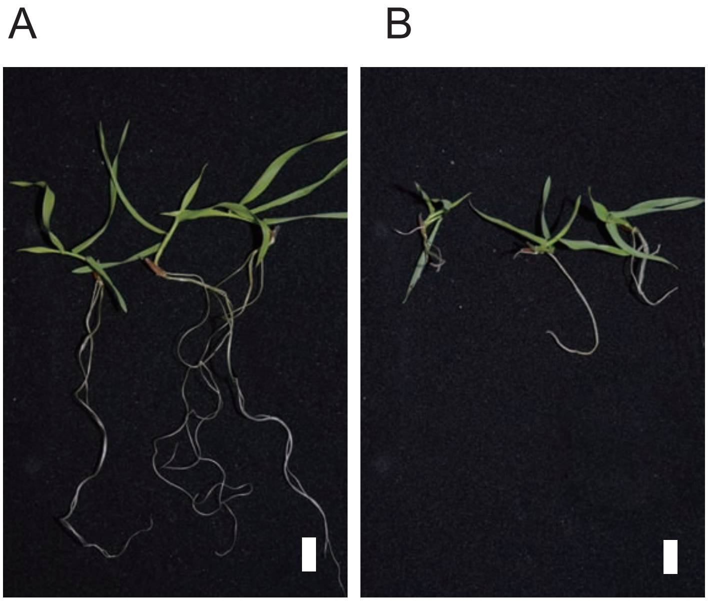

Figure S3. Images of ethylene-treated Brachyodium seedlings

Brachypodium seedlings were treated with a precursor of ethylene, ACC. A. Image of mock treated Brachypodium. B. Image of Brachypodium treated with 100 µM ACC for 1 week. Plants were germinated on 1/2 MS medium. Four-day-old Brachypodium plants were transplanted to liquid medium supplemented with or without ACC and cultured for one week with shaking. Scale bar = 1 cm.

Table S1 Comparison of treatments in Brachypodium, Arabidopsis and rice

Hormones	Chemicals	Arabidopsis (Goda 2008)	Brachypodium (This paper)	Rice (Garg 2012)	Rice (Sato 2012)
Auxin	IAA	1 µM	10 µM	50 µM	10 µM
Cytokinin	tZ	1 µM	1 µM		
	benzyl aminopurine			50 µM	1 µM
Gibberellin	GA4	5 µM(ga1)	3 µM	not available	
	GA3	1 µM(Col-0)		not available	10 µM
	Phx	10 µM	100 µM	not available	not available
Brassinosteroid	BL	10nM(Col-0)	1 µM	not available	1 µM
	BL	10nM(det2)			
	Brz220	10 µM	100 µM	not available	not available
Salicylic acid	SA	10 µM	100 µM	100 µM	
Abscisic acid	ABA	10 µM	10 µM	100 µM	50 µM
Jasmonate	MJ	10 µM	30 µM	100 µM	
	JA				100 µM
Ethylene	ACC	10 µM	100 µM	100 µM	not available

Table S2-1. GOE analysis of DEGs obtained under high-stringency conditions with InterProScan

GOE in Auxin	Term	P-value	FDR
GO:0031326	Regulation of cellular biosynthetic process	7.90E-12	2.30E-10
GO:0045449	Regulation of transcription	6.60E-12	2.30E-10
GO:0019219	Regulation of nucleobase, nucleoside, nucleotide and nucleic acid metabolic process	7.50E-12	2.30E-10
GO:0010468	Regulation of gene expression	9.20E-12	2.30E-10
GO:0009889	Regulation of biosynthetic process	7.90E-12	2.30E-10

GOE in CK	Term	P-value	FDR
GO:0000160	Two-component signal transduction system (phosphorelay)	9.20E-10	0.00000013
GO:0006807	Nitrogen compound metabolic process	1.70E-05	0.0012
GO:0032774	RNA biosynthetic process	1.70E-04	0.0034
GO:0065007	Biological regulation	1.10E-04	0.0034
GO:0051252	Regulation of RNA metabolic process	1.20E-04	0.0034

GOE in SA	Term	P-value	FDR
GO:0080090	Regulation of primary metabolic process	0.048	0.52
GO:0019222	Regulation of metabolic process	0.057	0.52
GO:0031326	Regulation of cellular biosynthetic process	0.036	0.52
GO:0031323	Regulation of cellular metabolic process	0.04	0.52
GO:0045449	Regulation of transcription	0.035	0.52

GOE in ABA	Term	P-value	FDR
GO:0050794	Regulation of cellular process	6.1E-11	7.1E-09
GO:0051252	Regulation of RNA metabolic process	7.4E-11	7.1E-09
GO:0006355	Regulation of transcription, DNA-dependent	7.4E-11	7.1E-09
GO:0009415	Response to water	1.7E-10	9.8E-09
GO:0050789	Regulation of biological process	1.5E-10	9.8E-09

GOE in JA	Term	P-value	FDR
GO:0019752	Carboxylic acid metabolic process	0.000000012	0.0000011
GO:0043436	Oxoacid metabolic process	0.000000012	0.0000011
GO:0042180	Cellular ketone metabolic process	0.000000013	0.0000011
GO:0006082	Organic acid metabolic process	0.000000014	0.0000011
GO:0008152	Metabolic process	0.000000061	0.0000041

Table S2-2. GOE analysis of DEGs obtained under low-stringency conditions with InterProScan

GOE in Phx	Term	P-value	FDR
GO:0006979	Response to oxidative stress	2.90E-23	5.70E-21
GO:0042221	Response to chemical stimulus	7.90E-20	7.80E-18
GO:0006950	Response to stress	1.10E-16	7.40E-15
GO:0050896	Response to stimulus	3.30E-15	1.60E-13
GO:0051179	Localization	1.20E-03	3.30E-02

GOE in Brz	Term	P-value	FDR
GO:0006091	Generation of precursor metabolites and energy	6.80E-06	3.40E-04
GO:0006412	Translation	7.20E-05	1.80E-03
GO:0044249	Cellular biosynthetic process	1.70E-03	2.90E-02
GO:0009058	Biosynthetic process	3.20E-03	3.80E-02
GO:0009059	Macromolecule biosynthetic process	0.0046	0.038

GOE in Ethylene	Term	P-value	FDR
GO:0031326	Regulation of cellular biosynthetic process	0.00084	0.012
GO:0031323	Regulation of cellular metabolic process	0.00098	0.012
GO:0045449	Regulation of transcription	0.0008	0.012
GO:0019219	Regulation of nucleobase, nucleoside, nucleotide and nucleic acid metabolic process	0.00083	0.012
GO:0010468	Regulation of gene expression	0.00088	0.012

Table S3 GOE analysis of phytohormone-regulated genes in Arabidopsis

GOE in Auxin 3h	TERM	ONTOLOGY	pValue
GO:0009733	response to auxin stimulus	BP	4.03E-12
GO:0009719	response to endogenous stimulus	BP	1.03E-09
GO:0009725	response to hormone stimulus	BP	3.24E-09
GO:0010583	response to cyclopentenone	BP	3.56E-08
GO:0010033	response to organic substance	BP	8.16E-08

GOE in t-zeatin 1h	TERM	ONTOLOGY	pValue
GO:0009736	cytokinin mediated signaling pathway	BP	1.39E-06
GO:0071368	cellular response to cytokinin stimulus	BP	1.39E-06
GO:0009735	response to cytokinin stimulus	BP	4.28E-06
GO:0000156	phosphorelay response regulator activity	MF	8.86E-05
GO:0009628	response to abiotic stimulus	BP	0.0001746

GOE in SA 3h	TERM	ONTOLOGY	pValue
GO:0009627	systemic acquired resistance	BP	1.03E-36
GO:0009814	defense response, incompatible interaction	BP	2.55E-36
GO:0045087	innate immune response	BP	1.02E-34
GO:0006955	immune response	BP	1.38E-34
GO:0002376	immune system process	BP	6.08E-33

GOE in ABA 3h	TERM	ONTOLOGY	pValue
GO:0009415	response to water stimulus	BP	8.77E-27
GO:0009414	response to water deprivation	BP	1.40E-26
GO:1901700	response to oxygen-containing compound	BP	1.50E-22
GO:0009737	response to abscisic acid stimulus	BP	4.84E-22
GO:0097305	response to alcohol	BP	3.88E-21

GOE in MJA 3h	TERM	ONTOLOGY	pValue
GO:0009694	jasmonic acid metabolic process	BP	2.94E-42
GO:0009695	jasmonic acid biosynthetic process	BP	3.12E-37
GO:0009611	response to wounding	BP	1.00E-33
GO:0009753	response to jasmonic acid stimulus	BP	1.89E-32
GO:0044283	small molecule biosynthetic process	BP	4.81E-31

Table S4 Expression of GA-synthetic genes in Brachypodium

GeneID	Annotation	log2 cpm
Bradi1g56200	AT4G25420.1 | Symbols: GA5, GA20OX1, AT2301, ATGA20OX1 | 2-oxoglutarate (2OG) and Fe(II)-dependent oxygenase superfamily protein	-2.76
Bradi1g56210	AT5G51810.1 | Symbols: GA20OX2, AT2353, ATGA20OX2 | gibberellin 20 oxidase 2	-2.76
Bradi1g56220	AT4G25420.1 | Symbols: GA5, GA20OX1, AT2301, ATGA20OX1 | 2-oxoglutarate (2OG) and Fe(II)-dependent oxygenase superfamily protein	-2.76
Bradi2g16727	AT1G30040.1 | Symbols: ATGA2OX2, GA2OX2 | gibberellin 2-oxidase	-2.76
Bradi4g23540	AT4G21690.1 | Symbols: ATGA3OX3, GA3OX3 | gibberellin 3-oxidase 3	-2.76
Bradi2g16750	AT1G30040.1 | Symbols: ATGA2OX2, GA2OX2 | gibberellin 2-oxidase	-1.76
Bradi3g49390	AT4G21200.1 | Symbols: ATGA2OX8, GA2OX8 | gibberellin 2-oxidase 8	-1.56
Bradi2g32577	AT1G78440.1 | Symbols: ATGA2OX1, GA2OX1 | Arabidopsis thaliana gibberellin 2-oxidase 1	-1.04
Bradi2g24980	AT4G25420.1 | Symbols: GA5, GA20OX1, AT2301, ATGA20OX1 | 2-oxoglutarate (2OG) and Fe(II)-dependent oxygenase superfamily protein	-0.73
Bradi5g16040	AT4G21200.1 | Symbols: ATGA2OX8, GA2OX8 | gibberellin 2-oxidase 8	-0.47
Bradi1g59570	AT4G21200.1 | Symbols: ATGA2OX8, GA2OX8 | gibberellin 2-oxidase 8	-0.46
Bradi2g06670	AT1G78440.1 | Symbols: ATGA2OX1, GA2OX1 | Arabidopsis thaliana gibberellin 2-oxidase 1	-0.35
Bradi2g57027	AT4G25420.1 | Symbols: GA5, GA20OX1, AT2301, ATGA20OX1 | 2-oxoglutarate (2OG) and Fe(II)-dependent oxygenase superfamily protein	-0.28
Bradi2g34837	AT1G02400.1 | Symbols: ATGA2OX4, ATGA2OX6, DTA1, GA2OX6 | gibberellin 2-oxidase 6	0.16
Bradi2g19900	AT1G78440.1 | Symbols: ATGA2OX1, GA2OX1 | Arabidopsis thaliana gibberellin 2-oxidase 1	0.50
Bradi2g50280	AT1G30040.1 | Symbols: ATGA2OX2, GA2OX2 | gibberellin 2-oxidase	1.07
Bradi2g12440	AT1G02400.1 | Symbols: ATGA2OX4, ATGA2OX6, DTA1, GA2OX6 | gibberellin 2-oxidase 6	1.22
Bradi2g04840	AT4G21690.1 | Symbols: ATGA3OX3, GA3OX3 | gibberellin 3-oxidase 3	1.82
Bradi1g00950	AT4G25420.1 | Symbols: GA5, GA20OX1, AT2301, ATGA20OX1 | 2-oxoglutarate (2OG) and Fe(II)-dependent oxygenase superfamily protein	2.25

Table S5 GOE analysis of *ga1-5* mutant vs Col-0

GOID	TERM	ONTOLOGY	pValue
GO:0000041	transition metal ion transport	BP	4.66E-12
GO:0010167	response to nitrate	BP	1.59E-11
GO:0015698	inorganic anion transport	BP	4.78E-11
GO:0050896	response to stimulus	BP	5.72E-11
GO:0015706	nitrate transport	BP	7.61E-11
GO:0044711	single-organism biosynthetic process	BP	8.86E-11
GO:0044283	small molecule biosynthetic process	BP	2.90E-10
GO:0009987	cellular process	BP	1.28E-09
GO:0030001	metal ion transport	BP	1.86E-09
GO:0006811	ion transport	BP	1.04E-08
GO:0006820	anion transport	BP	1.34E-08
GO:0046165	alcohol biosynthetic process	BP	1.83E-08
GO:1901700	response to oxygen-containing compound	BP	2.40E-08
GO:0044281	small molecule metabolic process	BP	3.48E-08
GO:1901617	organic hydroxy compound biosynthetic process	BP	4.58E-08
GO:0042221	response to chemical stimulus	BP	6.95E-08
GO:0006694	steroid biosynthetic process	BP	1.18E-07
GO:0006812	cation transport	BP	1.18E-07
GO:0016126	sterol biosynthetic process	BP	1.30E-07
GO:0006066	alcohol metabolic process	BP	1.39E-07
GO:0044710	single-organism metabolic process	BP	1.81E-07
GO:0016125	sterol metabolic process	BP	2.11E-07
GO:0010106	cellular response to iron ion starvation	BP	3.22E-07
GO:1901615	organic hydroxy compound metabolic process	BP	3.31E-07
GO:0044765	single-organism transport	BP	4.10E-07
GO:0005975	carbohydrate metabolic process	BP	5.45E-07
GO:0006826	iron ion transport	BP	6.44E-07
GO:0008202	steroid metabolic process	BP	6.89E-07
GO:0009605	response to external stimulus	BP	7.23E-07
GO:0008152	metabolic process	BP	8.25E-07

Table S6-1 Expressions of expansins

	Gene IDs	log2 ratio	stat	cpm
EXPA	*AT4G38210-EXPA20*	-1.29	0.00	
	Bradi1g35830	-0.17	1.00	2.54
	AT3G03220-EXPA13	-0.17	0.19	
	Bradi5g19340	-0.17	1.00	5.16
	AT5G39310-EXPA24	-0.30	0.00	
	AT5G39300-EXPA25	#N/A	#N/A	
	AT5G39280-EXPA23	#N/A	#N/A	
	AT5G39290-EXPA26	#N/A	#N/A	
	AT5G39270-EXPA22	#N/A	#N/A	
	AT5G39260-EXPA21	-0.39	0.00	
	Bradi3g13720	-2.31	1.00	-2.46
	Bradi1g51990	0.24	1.00	2.96
	AT1G62980-EXPA18	0.48	0.18	
	AT1G12560-EXPA7	0.80	0.02	
	Bradi3g32070	0.00	1.00	-2.68
	Bradi3g43080	-2.31	1.00	-2.46
	Bradi2g10320	-0.15	1.00	2.65
	Bradi3g59460	-0.07	1.00	2.80
	Bradi1g61190	-0.34	1.00	-0.14
	Bradi2g53580	-0.08	1.00	5.80
	Bradi2g22290	-0.11	1.00	3.85
	AT2G40610-EXPA8	-1.86	0.01	
	AT5G05290-EXPA2	1.40	0.00	
	AT3G29030-EXPA5	-1.10	0.02	
	AT5G56320-EXPA14	-2.11	0.00	
	AT2G03090-EXPA15	-1.24	0.03	
	AT1G26770-EXPA10	-0.55	0.00	
	AT1G69530-EXPA1	0.00	0.99	
	AT5G02260-AT5G02260	0.09	0.58	
	AT2G37640-EXPA3	-0.36	0.06	
	AT2G28950-EXPA6	-0.77	0.08	
	AT3G55500-EXPA16	-0.43	0.00	
	AT2G39700-EXPA4	-1.02	0.00	
	Bradi1g03640	0.31	1.00	3.12
	Bradi1g74710	-0.45	1.00	-0.42
	Bradi3g27440	-0.05	1.00	2.37
	Bradi3g27450	0.04	1.00	2.62
	Bradi1g74740	3.20	1.00	-2.46
	Bradi3g27470	0.90	1.00	0.32

Table S6-2 Expressions of expansins

	Gene IDs	log2 ratio	stat	cpm
EXPA	*Bradi3g27460*	-0.34	1.00	2.86
	Bradi1g74720	0.18	1.00	1.87
	Bradi3g10100	0.00	1.00	-2.68
	Bradi3g09990	-0.03	1.00	0.68
	Bradi3g09940	0.37	1.00	0.71
	Bradi3g09967	0.00	1.00	-2.68
	Bradi3g19500	-0.08	1.00	2.25
	Bradi3g09960	0.62	1.00	1.41
	Bradi3g09950	1.29	1.00	-0.46
	Bradi3g09930	-0.12	1.00	-0.27
	AT1G20190-EXPA11	-0.52	0.39	
	Bradi1g74750	-0.39	1.00	0.16
	Bradi2g08760	-0.88	1.00	-0.15
	Bradi2g31760	-0.14	1.00	0.82
	Bradi5g04120	-0.56	1.00	1.62
	Bradi2g08780	-0.06	1.00	-0.46
	AT4G01630-EXPA17	-0.83	0.00	
	AT3G15370-EXPA12	-0.14	0.56	
EXPL	*AT4G17030-EXLB1*	0.07	0.76	
	Bradi1g26990	0.08	1.00	2.12
	AT4G38400-EXLA2	-0.15	0.00	
	AT3G45960-EXLA3	-1.23	0.00	
	AT3G45970-EXLA1	-0.34	0.60	
	Bradi1g28130	0.82	0.08	4.68
	Bradi3g32297	-0.26	1.00	2.19
	Bradi1g76270	-0.03	1.00	4.45
	Bradi1g76260	0.52	1.00	4.16
EXPB	*Bradi2g35007*	0.00	1.00	-2.68
	Bradi3g32810	0.00	1.00	-2.68
	Bradi4g00360	0.00	1.00	-2.68
	Bradi1g78120	0.00	1.00	-2.68
	Bradi3g33130	-0.09	1.00	5.18
	Bradi3g33140	0.06	1.00	4.34
	Bradi3g33120	0.29	1.00	1.81
	Bradi1g78340	-0.14	1.00	4.34
	Bradi3g33160	0.16	1.00	7.03
	Bradi1g78350	-0.06	1.00	6.79
	Bradi3g33110	-0.47	1.00	5.84
	Bradi3g33150	-0.23	1.00	7.16

Table S6-3 Expressions of expansins

	Gene IDs	log2 ratio	stat	cpm
EXPB	*AT1G65680-EXPB2*	-0.31	0.00	
	AT3G60570-EXPB5	-0.34	0.00	
	AT2G45110-EXPB4	#N/A	#N/A	
	Bradi3g50740	-0.25	1.00	5.06
	Bradi3g50750	-0.06	1.00	4.55
	Bradi5g17770	0.23	1.00	-1.18
	Bradi5g17780	0.00	1.00	-2.68
	Bradi4g41117	0.00	1.00	-2.68
	Bradi4g41110	0.00	1.00	-2.68
	Bradi1g13787	0.14	1.00	3.10
	Bradi5g17760	-0.52	1.00	-1.46
	Bradi3g50730	-4.84	1.00	-1.57
	AT4G28250-EXPB3	-0.94	0.01	
	AT2G20750-EXPB1	-1.14	0.01	
	Bradi3g49850	0.02	1.00	1.54
	Bradi5g16497	-0.21	1.00	1.73

Table S7 Primers for RT-PCR

Transcript	Most similar Arabidopsis or rice gene	Forward Primer	Reverse primer
Bradi4g02600.1	*IAA16*	GATGAACGGCATGAACGAGA	TCCTCATAGGTCGGAACGTACTC
Bradi2g31820.1	*IAA9*	GCTACCGCAAGAACACTATGGC	TTGACGTAGAGGAACCCCTGA
Bradi2g33417.1	*IAA18*	TCTGGCCTACAGCAATGGC	CGTCGACCACCTCTTCCG
Bradi5g55410.1	*LOC_Os02g57250*	TCAGAGGCCGAAACCAAGAA	TTCTCGCCTTCCTCCTTGTCT
Bradi1g69480.1	*ARR1*	GGATGAAGGTGCTGGTGGTAGA	CGCAGCATACTTAATGCTCTTGTG
Bradi2g61000.1	*ARR9*	GAAGGACATTCCAGTGGTGATCA	AACTCTTCGGCGCCATCTT
Bradi4g43090.2	*OsPR10*	GTCTGAGAACGTACCTTCCAGAATC	CTGATAGCCGTACCGGTTTCA
Bradi1g15030.1	*BR6ox2/CYP85A2*	TCAACCTTCCAGGAACAAGC	CCGCTTAAGAGAGCATCCAG
Bradi1g69040.1	*DWF4/CYP90B*	CTTGCTTAGCCTGCTCTTCG	GCGATCTCAAGATGCTCCTC
Bradi4g43110.1	*CPD/CYP90A*	GATGGTGGACTTCTGCCTGT	CGCCTTTCATGTTGGTCATA
Bradi1g11090.1	*GAI*	GGTACAAGGTGGAGGAGAAAGAC	GGATGTGTACTTACAGCGTTCAA
Bradi2g60750.1	*SCL3*	AATTTATTAGGCGTTTGGTCGAAT	CCATCCAGCAAAGTACTGGTAAC
Bradi2g22290.1	*AtEXP1*	ACTCCTACTTCAACCTGGTGCT	GTAGGTGTTGCTCTGCCAGTTCT
Bradi2g02050.1	*AT1G79520*	CAGGAGAGCCTTCTGAAGGATTT	GCACTGGTTTGATCCAAGCA
Bradi3g26920.1	*HsfA6B*	CCTCTTCAAGCACAGCAACTTCT	ACTCCCACCTGTCAGGATCAA
Bradi1g11280.1	*GLTP*	AACGACTGCGTCAAGAAGCA	GTCGTAGCAGCATCATACAACGTA
Bradi4g29360.1	*LOC_Os09g21120*	TTCTCTTCTACCTCTCGTCCAACG	GATGAGCTCCACCAGCTTCG
Bradi3g55730.1	*ERS2*	AACCAAATGGAGAGGGCTTT	TCATGCTCTCTGGGAGTCCT
Bradi3g57620.1	*EFE,ACO4*	AGGTTCTGGACCACGGCAT	TCGCGCACCCTCTTGTAGT
Bradi2g27140.1	*DL4170C*	CAACCAGTACCTCATTAACCATGTG	TCTGGTTCTCGTTGAACATCGA
Bradi5g00700.1	*OsETR2*	CAGTTCTGGAGGAGTCTCAGTTGA	CCATCATAGCTTCATGCTTTGC
Bradi4g27680.1	*LOC_Os01g73200*	ACGACCCCACCATGAACAA	GGTTCATCAGGTCGACGTAGTACTT
Bradi2g47550.1	*JMT*	GAAGGTAGCAGTGCGTGTATAGC	ACTCCACCAGTTGATACTGGAAA
Bradi3g37650.1	*OPR3*	GGGCGGCTGTTCATATCTAA	GGGACGGATAGTCGGTGTAA
Bradi3g34200.1	*OsMYC2*	CGACGCCATCTCTTACATCA	CCTCAATCTGGGAATGGAGA
Bradi1g57590.1	*PR1*	CGAGAAGAAGAACTACCACCATGAC	ACACCCGATGGCAGTCGA
Bradi2g44270.1	*WRKY70*	CAAATACGGCCAGAAGGACATC	GCACTTCTGGTCGTACTTGTGTG
Bradi2g52110.1	*AtMES1*	AGCTGCCTATTTCCATGCTGTT	ATCGAACCCACTCGCATCA
